# Constitutive Models for Dynamic Strain Aging in Metals: Strain Rate and Temperature Dependences on the Flow Stress †

**DOI:** 10.3390/ma13071794

**Published:** 2020-04-10

**Authors:** Yooseob Song, Daniel Garcia-Gonzalez, Alexis Rusinek

**Affiliations:** 1 Department of Civil Engineering, The University of Texas Rio Grande Valley, 1201 W University Dr, Edinburg, TX 78539, USA; 2 Department of Continuum Mechanics and Structural Analysis, University Carlos III of Madrid, Avda. de la Universidad 30, 28911 Leganés, Madrid, Spain; danigarc@ing.uc3m.es; 3 Laboratory of Microstructure Studies and Mechanics of Materials, UMR-CNRS 7239, Lorraine University, 7 rue Félix Savart, BP 15082, 57073 Metz CEDEX 03, France; alexis.rusinek@univ-lorraine.fr; 4 Chair of Excellence, Departamento de Ingeniería Mecánica, University Carlos III of Madrid, Avda. de la Universidad 30, 28911 Leganés, Madrid, Spain

**Keywords:** constitutive model, dynamic strain aging, probability function, strain rate effect, Q235B steel

## Abstract

A new constitutive model for Q235B structural steel is proposed, incorporating the effect of dynamic strain aging. Dynamic strain aging hugely affects the microstructural behavior of metallic compounds, in turn leading to significant alterations in their macroscopic mechanical response. Therefore, a constitutive model must incorporate the effect of dynamic strain aging to accurately predict thermo-mechanical deformation processes. The proposed model assumes the overall response of the material as a combination of three contributions: athermal, thermally activated, and dynamic strain aging stress components. The dynamic strain aging is approached by two alternative mathematical expressions: (i) model I: rate-independent model; (ii) model II: rate-dependent model. The proposed model is finally used to study the mechanical response of Q235B steel for a wide range of loading conditions, from quasi-static loading ($\dot{\varepsilon }=0.001\;s^{-1}$ and $\dot{\varepsilon }=0.02\;s^{-1}$) to dynamic loading ($\dot{\varepsilon }=800\;s^{-1}$ and $\dot{\varepsilon }=7000\;s^{-1}$), and across a broad range of temperatures (93 K−1173 K). The results from this work highlight the importance of considering strain-rate dependences (model II) to provide reliable predictions under dynamic loading scenarios. In this regard, rate-independent approaches (model I) are rather limited to quasi-static loading.

## 1. Introduction

From the macroscopic point of view, dynamic strain aging (DSA) can be described as an unexpected strengthening in the flow stress at a specific temperature range. The third-type strain aging effect (bell-shaped stress peak in stress-temperature graphs) and the Portevin–Le Chatelier (PLC) effect (related to serrated plastic flow stress) are different manifestations of DSA. In general, DSA is associated with spatio-temporal instabilities (as shown through serrated yielding and PLC bands), which implies that stress state is not homogeneous in the uniaxial experiment. Although serrated stresses were observed in some cases of the experiments in Wang et al. [1], the amplitude of the oscillation was small and Nemat-Nasser et al. [2] stated that it is only of second-order importance compared to the flow stress that is a measure of the total resistance force on moving dislocations. Therefore, in this work, only third-type strain aging (referred to as DSA hereafter for convenience) will be considered while building a constitutive model, and the PLC effect is not considered.

**Figure 1:**
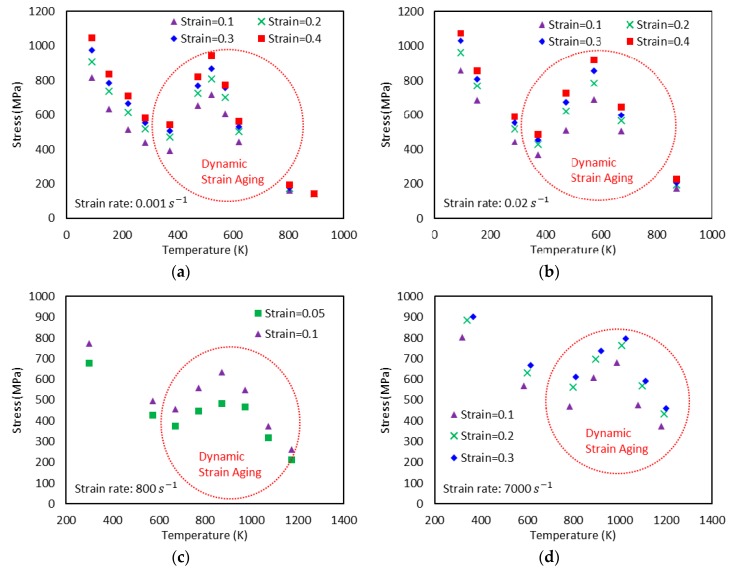
Experimental stress-temperature graphs for Q235B steel for different strain rates ($\dot{\varepsilon }$) and strain levels: (**a**) $\dot{\varepsilon }=0.001\;s^{-1}$, (**b**) $\dot{\varepsilon }=0.02\;s^{-1}$, (**c**) $\dot{\varepsilon }=800\;s^{-1}$, and (**d**) $\dot{\varepsilon }=7000\;s^{-1}$ [1]. Dynamic strain aging (DSA) is observed in all cases.

Generally, flow stress declines as temperature rises. However, at some combinations of strain rate and temperature, declining flow stress turns to an increase with temperature. In addition, further increase of temperature leads to another change of flow stress declining again after a peak is reached (see Figure 1). The height of this bell-shaped stress depends on the applied strain rate and strain level as shown in Figure 1. Moreover, the range of temperature where DSA becomes active is strongly dependent on the strain rate. DSA is hugely influenced by the crystal structure and even varies between metals with identical crystalline structure. Nemat-Nasser and coworkers have extensively studied the thermomechanical behaviors of body-centered cubic (bcc) and face-centered cubic (fcc) crystal structures focusing on DSA across a broad range of temperature and strain rate [3,4,5,6]. When it comes to niobium (bcc), DSA was detected only at quasi-static loading $\dot{\varepsilon }≅0.001/s$ within a temperature range of T≅450 K−700 K. On the contrary, no DSA was observed at higher strain rates $\dot{\varepsilon }≅0.001\;s^{-1}-10000\;s^{-1}$ for a wide temperature range T≅77 K−1000 K in tantalum (bcc), vanadium (bcc), and oxygen-free high thermal conductivity (OFHC) copper (fcc). DSA was also observed in various steel alloys, e.g., C45 [7], DH36 [8], and inconel 718 [9].

Q235B steel is used for a wide variety of applications due to its advanced characteristic in terms of strength, toughness, plasticity, and weldability. A systematic experimental DSA study on Q235B steel was conducted by [1] under quasi-static ($\dot{\varepsilon }=0.001\;s^{-1}$ and $\dot{\varepsilon }=0.02\;s^{-1}$) and dynamic loading ($\dot{\varepsilon }=800\;s^{-1}$ and $\dot{\varepsilon }=7000\;s^{-1}$) along with a wide range of temperature (93 K−1173 K) to address its plastic deformation mechanisms. Furthermore, the effect of strain rate on the DSA-induced hardening was discussed and incorporated in their constitutive model. Figure 1 shows the experimental stress-temperature responses presented by [1] for different strain rates and levels of strain. Note that DSA only becomes active when a specific range of temperature meets a specific range of strain rate. In their work, DSA was detected at 330 K≲T≲800 K for quasi-static loading and at 660 K≲T≲1300 K for dynamic loading.

In general, the interaction between diffusing solute atoms and mobile dislocations is identified as a key source of DSA [10]. From a physical point of view, DSA occurs when solute atoms are diffused to mobile dislocations that are temporarily trapped at the obstacle for a certain period before mobile dislocations travel to adjacent obstacles. DSA becomes active when the waiting time ($t_{w}$) of mobile dislocations corresponds with the aging time ($t_{a}$), which implies the effective time for the dislocation is aged. The waiting and the aging times are related to each other as follows: $dt_{a}/dt=1-t_{a}/t_{w}$ [11].

**Figure 2:**
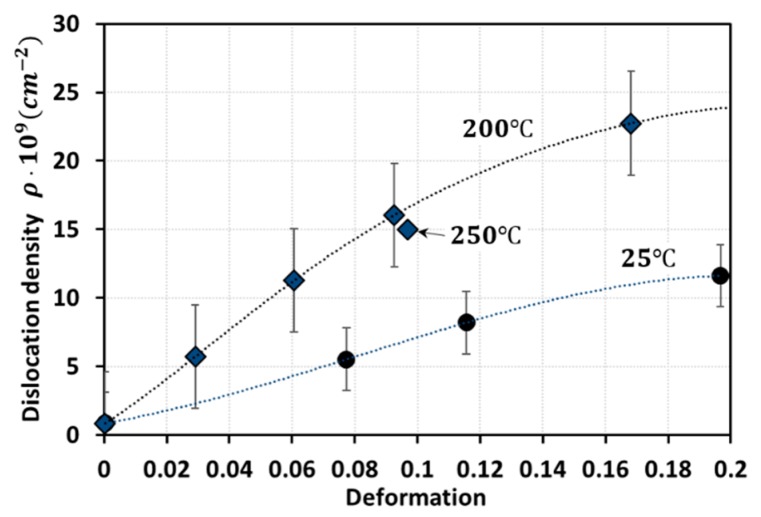
Dislocation density versus deformation graphs at different strain and temperature levels [12,13].

Generally, dislocation density tends to decline as temperature increases. However, the reverse was noted in the experiments published by [12], as well as the model predictions by [13] (see Figure 2). Following [13], the dislocation density can be given as a function of the equivalent plastic strain. The decomposition into athermal and thermal processes was also employed successfully by [14]. In addition, both experimental and analytical results show that the terms U−A and Ω are largely affected by the temperature variation during DSA (Figure 2). The term U represents the dislocation immobilization or annihilation rate, the term A represents the annihilation rate of the mobile dislocations, and the term Ω represents the probability of annihilation or re-mobilization of immobile dislocations. More details about these terms are presented in Section 2.2. This leads to the unpredicted result shown in Figure 2 where the dislocation densities at T=200 ℃ are larger than those at T=25 ℃.

The new constitutive model for Q235B steel developed in this work accounts, in a coupled formulation, for: thermal activation mechanics, dislocation interaction mechanics, decomposition of flow stress, and a mathematical description of the probability function. The model proposed is then calibrated and validated using available experimental data. All the derivations of the DSA ($\sigma_{D}$), thermal ($\sigma_{th}$), and athermal ($\sigma_{ath}$) components of the total flow stress (σ) rely on physical bases. To model the DSA phenomenon more accurately, the component $\sigma_{D}$ is assumed as a probability density-shaped function of the temperature T, the equivalent plastic strain $\varepsilon_{p}$, and its rate ${\dot{\varepsilon }}_{p}$ (refer to Section 2.2 for more details). This assumption is motivated on the dependences of the density of dislocations on the levels of temperature and plastic deformation, as shown in Figure 2. The Weibull distribution probability density function is used in this work to describe the DSA stress, $\sigma_{D}$.

The term ‘Voyiadjis–Abed (VA) model’ will be employed hereafter to indicate the constitutive model merely including the athermal and thermal components without DSA, i.e., $\sigma_{VA}=\sigma_{ath}+\sigma_{th}$ [15]. On the other hand, two mathematical models will be introduced for the variables in the DSA component $\sigma_{D}$, and they will be referred to as ‘proposed model I (or PM I)’ and ‘proposed model II (or PM II)’. The difference between these two models will be described in Section 2.2. The proposed models include all three elements, i.e., $\sigma_{PM\;I,\;II}=\sigma_{ath}+\sigma_{th}+\sigma_{D}$. PM I was originally proposed by [16] and PM II is newly proposed in this work. The VA model and PM I are expected to present some limitations to capture the DSA effect accurately. The main purpose of this work is to show the ability of PM II to overcome such limitations.

The DSA, thermal, and athermal components of the flow stress in the proposed models are formulated in Section 2. In Section 3, calibration is conducted taking the experimental data performed by [1] to obtain the material properties used in PM I and PM II. The stress-strain behaviors presented in Figure 2 will be reconsidered to investigate the DSA phenomenon in Section 4. The strain-rate sensitivity is discussed in Section 5.

## 2. Constitutive Models

A constitutive model without the DSA element naturally underestimates flow-stress values at the range of active DSA. This underestimation is even worse in dynamic manufacturing processes which may involve an increase in temperature. To construct a physically based constitutive model including physical characteristics of DSA, it is crucial to take into account the microstructural features of the materials as well as the dislocation dynamics, as was done by Klepaczko [17], Rusinek and Klepaczko [14], Voyiadjis, Song and Rusinek [16], Voyiadjis and Song [18], and Rusinek et al. [19]. In Section 2.1, the microstructurally/physically based formulation of the flow stress is derived in terms of athermal and thermal components. The DSA component is discussed in Section 2.2 based on [1,16,18].

The chemical composition (wt. %) of Q235B steel is as follows [1]: Mn (≤1.4), Si (≤0.35), C (0.17–0.22), S (≤0.045), P (≤0.045), Cu (≤0.03), Ni (≤0.03), Cr (≤0.03), and Fe (Bal.). Q235B steel is an alloy of these various elements with iron as the most dominant one. Therefore, the constitutive model developed in this work will combine both fcc and bcc approaches to define the plastic deformation behavior of Q235B steel in a wide range of strain rates and temperature. Iron has a characteristic phase transformation between bcc and fcc crystal structures according to the temperature range, however this will be ignored in this work.

In fcc metallic crystalline structures, the thermally activated mechanism is controlled and dominated by the long-range interactions related to heterogeneous microstructural occurrence and evolution of dislocations, which suggests a strong dependence on plastic strain. Strain rate and temperature do not affect the initial yield stress in fcc metals. It implies that the yielding points will be the same in the stress-strain graphs regardless of strain rate and temperature. In bcc metallic crystalline structures, on the other hand, the deformation mechanism is attributed to resistance of the dislocation motions by the short-range interactions (Peierls barriers) provided by the lattice itself. Therefore, the thermal yield stress in bcc metals is highly dependent on temperature and strain rate whereas hardening is hardly affected by either temperature or strain rate. These mechanisms will be reflected in the development of the proposed model.

### 2.1. Athermal and Thermal Stresses

Characteristics of metals during plastic deformation can be accurately modeled by investigating their dislocation dynamics including interaction, multiplication, and motion of dislocations.

The plastic shear strain rate ${\dot{\gamma }}^{p}$ is given as follows using Orowan’s equation:

(1) $${\dot{\gamma }}^{p}=b\rho_{m}v$$

where b denotes the Burgers vector, $\rho_{m}$ denotes the density of mobile dislocations, and v denotes the average velocity of mobile dislocations.

From [20], the following relation is assumed:

(2) $${\dot{\varepsilon }}_{ij}^{p}={\dot{\gamma }}^{p}M_{ij}$$

where ${\dot{\varepsilon }}_{ij}^{p}$ indicates the macroscale plastic strain rate tensor. The term $M_{ij}$ denotes the symmetric Schmidt orientation tensor, which is defined as follows:

(3) $$M_{ij}=\frac{1}{2}\left(n_{i}⊗s_{j}+s_{i}⊗n_{j}\right)$$

where the terms s and n are the unit vector in the slip direction and the unit normal vector on the slip plane, respectively.

Substituting Equation (1) into Equation (2) gives the following expression for the equivalent plastic strain rate ${\dot{\varepsilon }}_{p}$.

(4) $${\dot{\varepsilon }}_{p}=\sqrt{\frac{2}{3}{\dot{\varepsilon }}_{ij}^{p}{\dot{\varepsilon }}_{ij}^{p}}=\bar{m}b\rho_{m}v$$

where $\bar{m}=\sqrt{2M_{ij}M_{ij}/3}$ represents the Schmidt orientation factor.

Following [21], the variation of the dislocation density with respect to the equivalent plastic strain is given as follows:

(5) $$\frac{\partial \rho }{\partial \varepsilon_{p}}=M-k_{a}\left(\rho -\rho_{i}\right)$$

where the term $k_{a}$ represents the dislocation annihilation factor which depends on the strain rate and temperature. The term M is the multiplication factor defined as M=1/bl, where l is the dislocation mean free path. The terms $\rho_{i}$ and ρ represent the initial and total dislocation densities, respectively.

The average dislocation velocity v can be determined using the thermally activated mechanism [18]. Using the well-known Arrhenius equation [22], the following expression is used in this work for this term [20]:

(6) $$v=v_{0}\exp\left(-\frac{G}{kT}\right)$$

where the term $v_{0}=d/t_{w}$ denotes the referential velocity of a dislocation where d denotes the average traveling distance of a dislocation from obstacle to obstacle. The terms k and T denote the Boltzmann constant and temperature in Kelvin, respectively. The activation free energy G may be dependent on the internal structure as well as the shear stress. Following [23], one can relate the activation energy G to the thermal flow stress $\sigma_{th}$ as follows:

(7) $$G=G_{0}{\left(1-{\left(\frac{\sigma_{th}}{\hat{\sigma }}\right)}^{p}\right)}^{q}$$

where the superscripts p and q denote the thermal hardening parameters and the shape of the short-range barriers. The term $\hat{\sigma }$ represents the threshold stress ($\hat{\sigma }=\sigma_{th}$ when G=0) and $G_{0}$ represents the referential Gibbs energy.

Substituting Equations (5) and (6) into Equation (4) and using Equation (7), the thermal part $\sigma_{th}$ can be calculated as follows:

(8) $$\sigma_{th}=\hat{\sigma }{\left(1-{\left(\beta_{1}T-\beta_{2}T\ln\frac{{\dot{\varepsilon }}_{p}}{{\dot{\varepsilon }}_{p}^{0}}\right)}^{\frac{1}{q}}\right)}^{\frac{1}{p}}$$

where ${\dot{\varepsilon }}_{p}^{0}$ denotes the referential equivalent plastic strain rate.

Meanwhile, the terms $\beta_{1}$ and $\beta_{2}$ are defined, respectively, as follows:

(9) $$\beta_{1}=\frac{k}{G_{0}}\ln\left(\frac{\bar{m}b^{2}\rho_{m}v_{0}}{b-\bar{m}d\left(\lambda_{1}-b^{2}\lambda_{2}\rho_{m}-b\lambda_{3}\rho_{f}^{0.5}\right)}\right)$$

(10) $$\beta_{2}=\frac{k}{G_{0}}$$

where $\rho_{f}$ denotes the forest dislocation density. The coefficients $\lambda_{i}\;\left(i=1-3\right)$ are related to the immobilization [18]. Note that the parameter $\beta_{1}$ is assumed as a fixed value in this work and Equation (9) does not apply.

Broadly, there exists two kinds of barriers blocking the dislocations’ movement in the crystal lattice: the short-range barrier caused by the forest dislocations and the long-range barrier caused by the material structure. The former can be overcome using the thermal activation energy, whereas the latter cannot. As a result, the total flow stress (σ) is additively decomposed into the thermal ($\sigma_{th}$) and athermal ($\sigma_{ath}$) components as follows:

(11) $$\sigma =\sigma_{ath}+\sigma_{th}.$$

Several works have demonstrated that the assumption of additive decomposition is valid [2,6,24].

The athermal component $\sigma_{ath}\left(\varepsilon_{p}\right)$ is given as a function of the equivalent plastic strain $\varepsilon_{p}$. The thermal component $\sigma_{th}\left(\varepsilon_{p},\;{\dot{\varepsilon }}_{p},\;T\right)$ is composed of the bcc part and the fcc part as mentioned earlier in this work, i.e., $\sigma_{th}=\sigma_{th}^{bcc}+\sigma_{th}^{fcc}$. The bcc part ($\sigma_{th}^{bcc}$) is given as a function of ${\dot{\varepsilon }}_{p}$ and T and the fcc part ($\sigma_{th}^{fcc}$) is given as a function of $\varepsilon_{p}$, ${\dot{\varepsilon }}_{p}$, and T as follows:

(12) $$\sigma_{ath}\left(\varepsilon_{p}\right)=Y_{a}+B_{1}\varepsilon_{p}^{n_{1}}.$$

For bcc

(13) $$\sigma_{th}^{bcc}\left({\dot{\varepsilon }}_{p},\;T\right)=Y_{d}{\left(1-{\left(\beta_{1}T-\beta_{2}T\ln\frac{{\dot{\varepsilon }}_{p}}{{\dot{\varepsilon }}_{p}^{0}}\right)}^{\frac{1}{q}}\right)}^{\frac{1}{p}}$$

and for fcc

(14) $$\sigma_{th}^{fcc}\left(\varepsilon_{p},\;{\dot{\varepsilon }}_{p},\;T\right)=B_{2}\varepsilon_{p}^{n_{2}}{\left(1-{\left(\beta_{1}T-\beta_{2}T\ln\frac{{\dot{\varepsilon }}_{p}}{{\dot{\varepsilon }}_{p}^{0}}\right)}^{\frac{1}{q}}\right)}^{\frac{1}{p}}$$

where $Y_{a}$ denotes the athermal yield stress and the parameters $B_{1}$ and $n_{1}$ denote the athermal hardening parameters. The parameter $Y_{d}$ denotes the thermal yield stress and the terms $B_{2}$, $n_{2}$, p, and q denote the thermal hardening parameters.

The combination of the two parts gives the total thermally activated flow stress component as follows:

(15) $$\sigma_{th}\left(\varepsilon_{p},\;{\dot{\varepsilon }}_{p},\;T\right)=Y_{d}{\left(1-{\left(\beta_{1}^{Y}T-\beta_{2}^{Y}T\ln\frac{{\dot{\varepsilon }}_{p}}{{\dot{\varepsilon }}_{p}^{0}}\right)}^{\frac{1}{q}}\right)}^{\frac{1}{p}}+B_{2}\varepsilon_{p}^{n_{2}}{\left(1-{\left(\beta_{1}^{H}T-\beta_{2}^{H}T\ln\frac{{\dot{\varepsilon }}_{p}}{{\dot{\varepsilon }}_{p}^{0}}\right)}^{\frac{1}{q}}\right)}^{\frac{1}{p}}.$$

The material parameters for Q235B to define $\sigma_{ath}$ and $\sigma_{th}$ are determined in Section 3. In addition, high deformation rates can result in inelastic dissipation leading to local temperature increment by means of adiabatic heating. In such dynamic scenarios, the effect of thermal softening due to temperature evolution during the deformation process is considered in the thermal component in Equation (15). Note that, at quasi-static loading conditions, inelastic heating is dissipated by conduction and convection terms and, therefore, isothermal conditions can be assumed during the deformation process. The increment in temperature during dynamic deformation arising from adiabatic heating can be computed as follows [15]:

(16) $$\Delta T=\frac{\beta }{c_{p}\bar{\rho }}\overset{\varepsilon_{p}}{\underset{0}{\int }}\sigma d\varepsilon_{p}$$

where $\bar{\rho }$ represents the material density and $c_{p}$ represents the specific heat at constant pressure. In this work, the Taylor–Quinney empirical coefficient β is defined as 0.9, as commonly assumed for most metals [25]. Making use of Equation (16), the temperature is updated during the plastic deformation process to account for adiabatic heating. Note that the effect of thermal softening on the flow stress is only considered for dynamic loading ($\dot{\varepsilon }=800\;s^{-1}$ and $7000\;s^{-1}$). On the contrary, isothermal conditions are assumed for quasi-static loading ($\dot{\varepsilon }=0.001\;s^{-1}$ and $0.02\;s^{-1}$).

### 2.2. DSA-Induced Stress

The relationship between the dislocation density and the equivalent plastic strain is given as follows [13]:

(17) $$\frac{d\rho }{d\varepsilon_{p}}=U-A-\Omega \rho .$$

From Equation (17), the dislocation density ρ can be obtained by:

(18) $$\rho =\frac{U-A}{\Omega }\left[1-\exp\left(-\Omega \varepsilon_{p}\right)\right]+\rho_{0}\exp\left(-\Omega \varepsilon_{p}\right)$$

where $\rho_{0}$ denotes the initial dislocation density.

**Figure 3:**
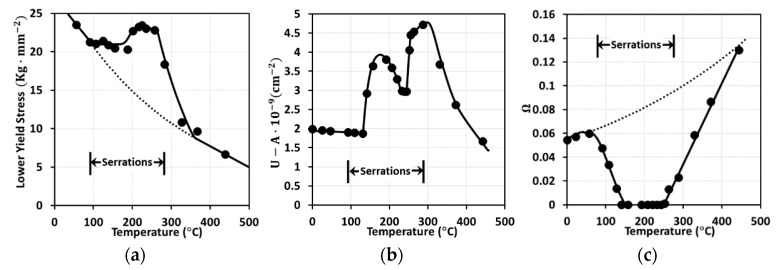
Profiles of model predictions (lines) and experimental data (dots) according to the temperature variation: (**a**) lower yield stress, (**b**) U−A and (**c**) Ω [13].

Bergstrom and Roberts [13] demonstrated the existence of the DSA phenomenon through experiments and model predictions as shown in Figure 3. It was observed that the level of yield stress ($\sigma =\alpha \mu b\sqrt{\rho }$ with α being a material constant and μ the shear modulus) in the dislocation model of Taylor [26] increases at a certain range of temperature due to the large value of U−A and the low value of Ω. Substituting Equation (18) into $\sigma =\alpha \mu b\sqrt{\rho }$ results in the following flow stress:

(19) $$\sigma =\sigma_{0}+\alpha \mu b{\left\{\frac{U-A}{\Omega }\left[1-\exp\left(-\Omega \varepsilon_{p}\right)\right]+\rho_{0}\exp\left(-\Omega \varepsilon_{p}\right)\right\}}^{1/2}$$

where $\sigma_{0}$ denotes the strain-independent friction stress. Consequently, one can conclude that DSA may be characterized using a probability function capturing the probabilistic nature of the physical phenomenon.

An extra term $\sigma_{D}$ in the form of probability function is introduced to model the bell-shaped hardening due to DSA. By assuming $\sigma_{D}\left(\varepsilon_{p},\;{\dot{\varepsilon }}_{p},\;T\right)$, the proposed model is formed as follows:

(20) $$\sigma_{PM\;I,\;II}\left(\varepsilon_{p},\;{\dot{\varepsilon }}_{p},\;T\right)=\sigma_{ath}\left(\varepsilon_{p}\right)+\sigma_{th}\left(\varepsilon_{p},\;{\dot{\varepsilon }}_{p},\;T\right)+\sigma_{D}\left(\varepsilon_{p},\;{\dot{\varepsilon }}_{p},\;T\right)$$

where the two components $\sigma_{ath}$ and $\sigma_{th}$ are shown, respectively, by Equations (12) and (15).

In this work, two mathematical models are examined for $\sigma_{D}$. In the first model (proposed model, PM I), the strain-rate effect on the magnitude of DSA-induced hardening (i.e., height of bell-shaped stress) is not included. This model was used in the authors’ previous works [16,18]. However, in [1] it was observed that the temperature range of active DSA shifts to more elevated temperatures and the magnitude of DSA decreases with strain rate, which implies that strain rate strongly affects the height of DSA-induced hardening. This observation is incorporated in the second model (proposed model II, PM II), whose comparison with the first approach (PM I) will be presented.

#### 2.2.1. Proposed Model I (PM I)

To characterize $\sigma_{D}$, the following standard parametrization formulation of the Weibull distribution probability density function was used in [16,18]:

(21) $$\sigma_{D}\left(\varepsilon_{p},\;{\dot{\varepsilon }}_{p},\;T\right)=a_{D}\left(\varepsilon_{p}\right)exp\left[-\frac{{\left\{T-W\left({\dot{\varepsilon }}_{p}\right)\right\}}^{2}}{b_{D}\left(\varepsilon_{p}\right)}\right]$$

where both the shape and scale of $\sigma_{D}$ are determined by the terms $a_{D}>0$ and $b_{D}>0$. The term W denotes the temperature corresponding to the flow stress peak at which the interaction between dislocations and solute atoms becomes the strongest. The term $b_{D}$ reflects the temperature range of DSA. In this work, a power-law form is employed for the functional expressions of $a_{D}$, $b_{D}$, and W, i.e., $a_{D}\left(\varepsilon_{p}\right)=k_{a}\varepsilon_{p}^{n_{a}}$, $b_{D}\left(\varepsilon_{p}\right)=k_{b}\varepsilon_{p}^{n_{b}}$, and $W\left({\dot{\varepsilon }}_{p}\right)=k_{W}{\dot{\varepsilon }}_{p}^{n_{W}}$, although other types of functional expression are also applicable as done by [16] and [18]. The constants ($k_{a}$, $k_{b}$, and $k_{W}$) and the law’s exponents ($n_{a}$, $n_{b}$, and $n_{W}$) are determined in Section 3 using the experimental data.

#### 2.2.2. Proposed Model II (PM II)

Wang, Guo, Gao, and Su [1] also used the identical form of the function presented in Equation (21) to model the DSA effect. However, the difference is made in the terms $a_{D}$, $b_{D}$, and W. They are all defined as functions of not only $\varepsilon_{p}$ but also ${\dot{\varepsilon }}_{p}$, i.e., $a_{D}\left(\varepsilon_{p},\;{\dot{\varepsilon }}_{p}\right)$, $b_{D}\left(\varepsilon_{p},\;{\dot{\varepsilon }}_{p}\right)$, and $W\left(\varepsilon_{p},\;{\dot{\varepsilon }}_{p}\right)$, as follows:

(22) $$a_{D}\left(\varepsilon_{p},\;{\dot{\varepsilon }}_{p}\right)=\left({\bar{a}}_{D}ln\frac{{\dot{\varepsilon }}_{p}}{\dot{\zeta }}+{\overset{=}{a}}_{D}\right)\varepsilon_{p}^{n_{3}}$$

(23) $$b_{D}\left(\varepsilon_{p},\;{\dot{\varepsilon }}_{p}\right)={\left(\frac{T_{2}}{ln\frac{{\dot{\varepsilon }}_{p}}{\dot{\zeta }}-\eta ln\frac{\varepsilon_{p}}{\varepsilon_{p}^{0}}}\right)}^{2}$$

(24) $$W\left(\varepsilon_{p},\;{\dot{\varepsilon }}_{p}\right)=\frac{T_{1}}{ln\frac{{\dot{\varepsilon }}_{p}}{\dot{\zeta }}-\eta ln\frac{\varepsilon_{p}}{\varepsilon_{p}^{0}}}$$

where the material constants ${\bar{a}}_{D}$, ${\overset{=}{a}}_{D}$, $\dot{\zeta }$, $n_{3}$, $T_{1}$, $T_{2}$, η, and $\varepsilon_{p}^{0}$ are calibrated using experimental data. Derivation of Equations (22)–(24) are given in detail by [1].

## 3. Model Validation and Calibration

### 3.1. Athermal and Thermal Stressesl

**Figure 4:**
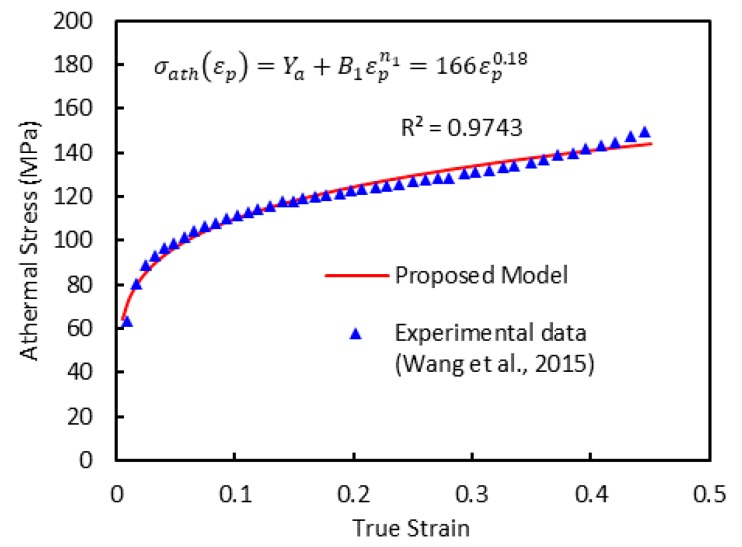
The athermal flow stress-strain curve from the experiments [1] and the proposed model.

As a first step for the model calibration, the stress-temperature curves for different plastic strain-level and strain-rate conditions are used. The general tendency consists of firstly a decrease of flow stress with temperature up to a critical temperature value. From this point, the flow stress keeps almost constant with temperature. The constant level of stress at that specific temperature indicates the athermal flow stress, $\sigma_{ath}$. The material parameters ($Y_{a}$, $B_{1}$, and $n_{1}$) in Equation (12) can be determined using the experimental data provided by [1]. The parameter $Y_{a}$ indicates the athermal flow stress at the initial yield ($\varepsilon_{p}=0$), in other words, the elastic part. In this work, this parameter is set as zero since the efforts of the current study aim to address the plastic model. Figure 4 shows a comparison between experimental data and model predictions (after calibration of material parameters) by means of athermal stress-strain curves.

Meanwhile, the thermal flow stress $\sigma_{th}$ is computed using the relation $\sigma_{th}=\sigma -\sigma_{ath}$ excluding the DSA component. The thermal degradation mechanism can be suitably captured by choosing the appropriate values of p and q. The following range of values are used for this purpose: 0≤p≤1 and 1≤q≤2. In this work, the values of p=0.51 and q=1.65 are used. To obtain the thermal yield stress $Y_{d}$, the flow stress at initial yield point $\sigma_{\varepsilon_{p}=0}$ is employed. Using Equations (12) and (15), $Y_{d}$ can be obtained by plotting ${\left(\sigma_{\varepsilon_{p}=0}-Y_{a}\right)}^{p}$ versus $T^{\frac{1}{q}}$ for each strain rate. The ${\left(1-{\left(\left(\sigma_{\varepsilon_{p}=0}-Y_{a}\right)/Y_{d}\right)}^{p}\right)}^{q}$ versus ${\dot{\varepsilon }}_{p}$ graphs at certain temperatures are used to determine $\beta_{1}^{Y}$ and $\beta_{2}^{Y}$. Similarly, the ${\left(\sigma -Y_{d}{\left(1-{\left(\beta_{1}^{Y}T-\beta_{2}^{Y}T\frac{{\dot{\varepsilon }}_{p}}{{\dot{\varepsilon }}_{p}^{0}}\right)}^{1/q}\right)}^{1/p}-Y_{a}-B_{1}\varepsilon_{p}^{n_{1}}\right)}^{p}$ versus $T^{\frac{1}{q}}$ graphs at different levels of plastic strain along with certain strain rate are plotted to determine $B_{2}$ and $n_{2}$. Lastly, the ${\left(1-{\left(\left(\sigma -Y_{d}{\left(1-{\left(\beta_{1}^{Y}T-\beta_{2}^{Y}T\frac{{\dot{\varepsilon }}_{p}}{{\dot{\varepsilon }}_{p}^{0}}\right)}^{1/q}\right)}^{1/p}-Y_{a}-B_{1}\varepsilon_{p}^{n_{1}}\right)/B_{2}\varepsilon_{p}^{n_{2}}\right)}^{p}\right)}^{q}$ versus ${\dot{\varepsilon }}_{p}$ graphs at certain plastic strain and temperature are plotted to determine $\beta_{1}^{H}$ and $\beta_{2}^{H}$.

**Figure 5:**
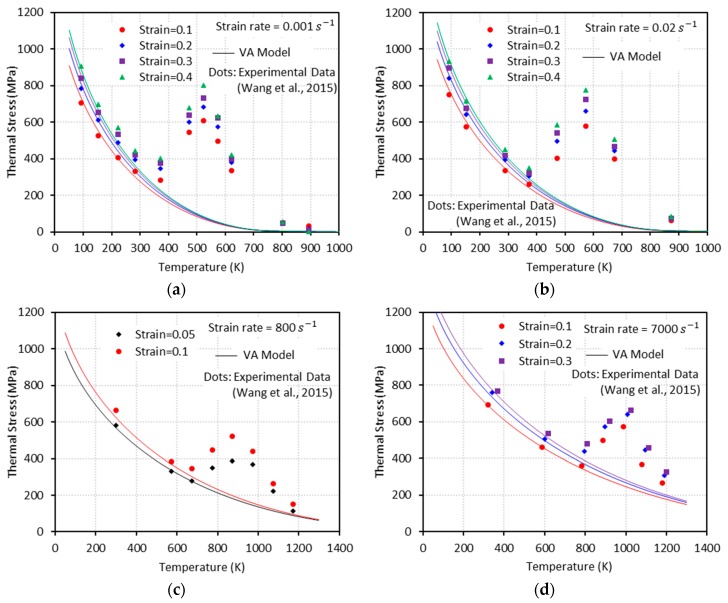
The thermal flow stress versus temperature curves from the experiments [1] and the VA model (Equation (15)) with (**a**) $\dot{\varepsilon }=0.001\;s^{-1}$, (**b**) $\dot{\varepsilon }=0.02\;s^{-1}$, (**c**) $\dot{\varepsilon }=800\;s^{-1}$, and (**d**) $\dot{\varepsilon }=7000\;s^{-1}$.

Following the above mentioned procedure, the material parameters associated to the thermal flow stresses $\sigma_{th}$ are calibrated by comparison with experimental data. In this regard, Figure 5 gives the comparison between the experimental data and the model predictions accounting for temperature, strain, and strain-rate dependences. These results show a good predictive capability of the model, given by Equation (15), to describe the thermally activated component of the flow stress. The experimental data in these figures were obtained by subtracting the athermal stress (Figure 4) from the total stress (Figure 1). The athermal- and thermal-related material parameters for Q235B steel used in the VA model are summarized in Table 1.

### 3.2. DSA-Induced Stress

Next, one needs to define the proper expressions for $a_{D}$, $b_{D}$, and W to capture the DSA-induced flow stress. The bell-shaped DSA-induced flow stresses observed in a series of experiments with $\dot{\varepsilon }=0.001\;s^{-1}$, $0.02\;s^{-1}$, $800\;s^{-1}$, and $7000\;s^{-1}$ conducted by [1] (Figure 1) are used to define these expressions. A negligibly small elastic range is assumed in this work ($\varepsilon =\varepsilon_{p}$). Therefore, the following assumption is made: $\dot{\varepsilon }={\dot{\varepsilon }}_{p}$.

#### 3.2.1. Proposed Model I (PM I)

**Figure 6:**
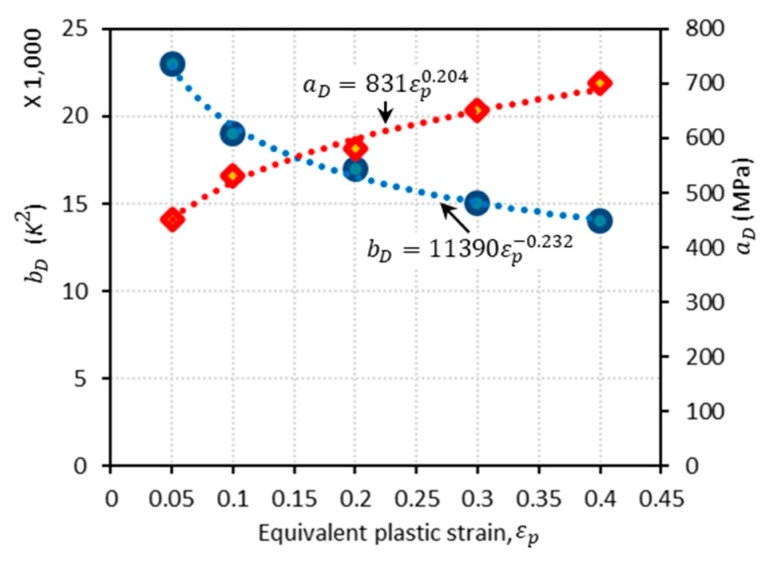
The plots of $a_{D}$ and $b_{D}$ versus $\varepsilon_{p}$. Dots for both of the parameters are obtained from the experimental data [1]. The corresponding trend lines are displayed using a power law form.

From the comparison between the experimental data and the DSA component of flow stress computed by Equation (21), the parameters $a_{D}$ and $b_{D}$ are easily determined, as revealed in Figure 6. More details about how to determine their functional form are provided in the authors’ previous works [16,18]. The expressions for $a_{D}$ and $b_{D}$ are given, respectively, as a function of the equivalent plastic strain by:

(25) $$a_{D}\left(\varepsilon_{p}\right)=831\varepsilon_{p}^{0.204}\;\left(MPa\right)$$

(26) $$\mathrm{and}\;b_{D}\left(\varepsilon_{p}\right)=11390\varepsilon_{p}^{-0.232}\;\left(K^{2}\right).$$

**Figure 7:**
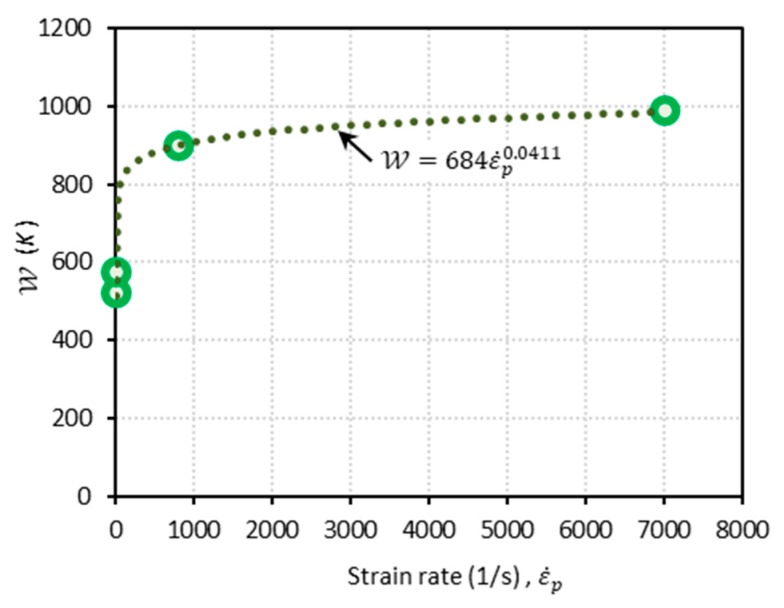
The plot of W versus ${\dot{\varepsilon }}_{p}$. Dots for both of the parameters are obtained from the experimental data [1]. The corresponding trend lines are displayed using a power law form.

Meanwhile, the function W depends on the strain rate. Following a similar procedure to obtain $a_{D}$ and $b_{D}$, the following expression for W is determined (see Figure 7):

(27) $$W\left({\dot{\varepsilon }}_{p}\right)=684{\dot{\varepsilon }}_{p}^{0.0411}\;\left(K\right)$$

Substituting Equations (25)–(27) into Equation (21) gives the following formulation of the DSA-induced flow stress $\sigma_{D}$ for PM I.

(28) $$\sigma_{D}{}_{PM\;I}\left(\varepsilon_{p},\;{\dot{\varepsilon }}_{p},\;T\right)=831\varepsilon_{p}^{0.204}\exp\left[-\frac{{\left\{T-684{\dot{\varepsilon }}_{p}^{0.0411}\right\}}^{2}}{11390\varepsilon_{p}^{-0.232}}\right].$$

**Figure 8:**
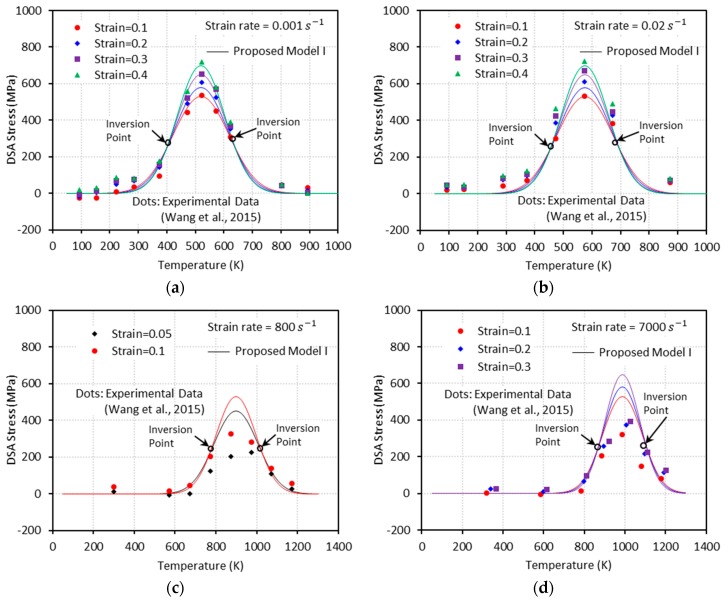
The DSA-induced flow stress versus temperature curves from the experiments [1] and the proposed model I with (**a**) $\dot{\varepsilon }=0.001\;s^{-1}$, (**b**) $\dot{\varepsilon }=0.02\;s^{-1}$, (**c**) $\dot{\varepsilon }=800\;s^{-1}$, and (**d**) $\dot{\varepsilon }=7000\;s^{-1}$.

The bell-shaped DSA-induced hardening versus temperature graphs are shown in Figure 8 at the designated strain levels. PM I is found able to capture the experimental measurements under quasi-static loading, cases (a) and (b). However, this model does not provide reliable predictions under dynamic loading, cases (c) and (d). This predictive limitation of the PM I can be explained by the lack of strain-rate effect consideration on the magnitude of DSA-induced hardening. In addition, the inversion of the bell-shaped hardening is detected in all cases in the initial and final stages of DSA, which is physically unreasonable.

**Figure 9:**
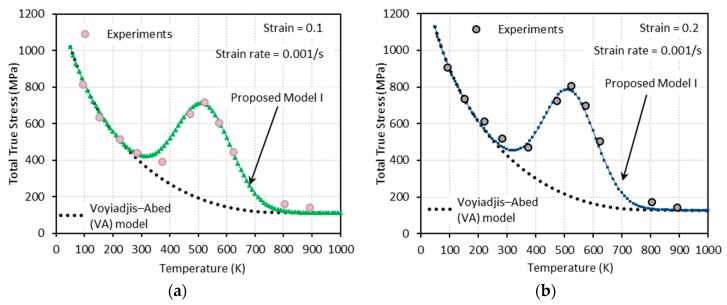

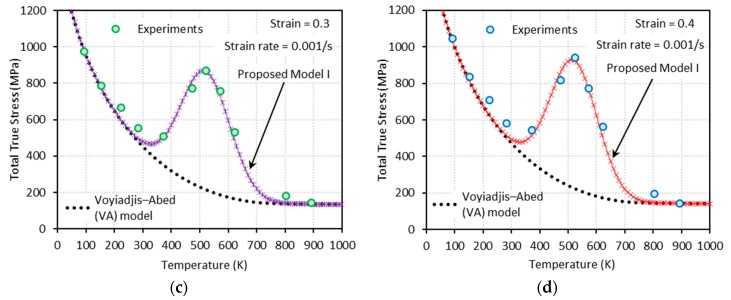
Comparisons between model predictions from the VA and proposed model I and experimental data from [1] on the total true stress versus temperature responses at (**a**) ε=0.1, (**b**) ε=0.2, (**c**) ε=0.3, and (**d**) ε=0.4. Quasi-static loading with $\dot{\varepsilon }=0.001\;s^{-1}$ is applied.

**Figure 10:**
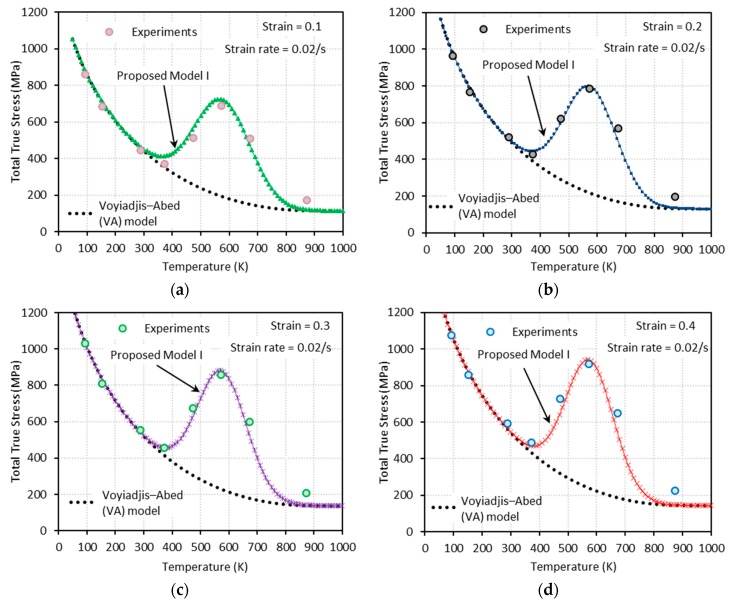
Comparisons between model predictions from the VA and proposed model I and experimental data from [1] on the total true stress versus temperature responses at (**a**) ε=0.1, (**b**) ε=0.2, (**c**) ε=0.3. and (**d**) ε=0.4. Quasi-static loading with $\dot{\varepsilon }=0.02\;s^{-1}$ is applied.

**Figure 11:**
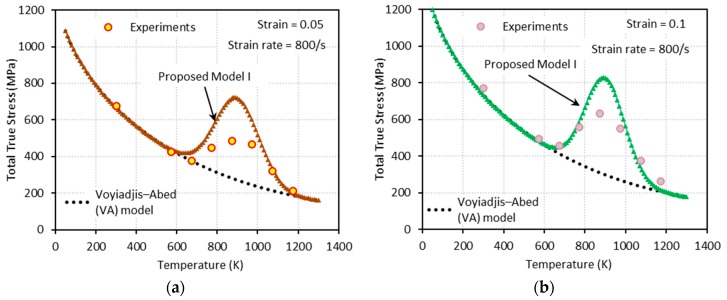
Comparisons between model predictions from the VA and proposed model I and experimental data from [1] on the total true stress versus temperature responses at (**a**) ε=0.05 and (**b**) ε=0.1. Dynamic loading with $\dot{\varepsilon }=800\;s^{-1}$ is applied.

**Figure 12:**
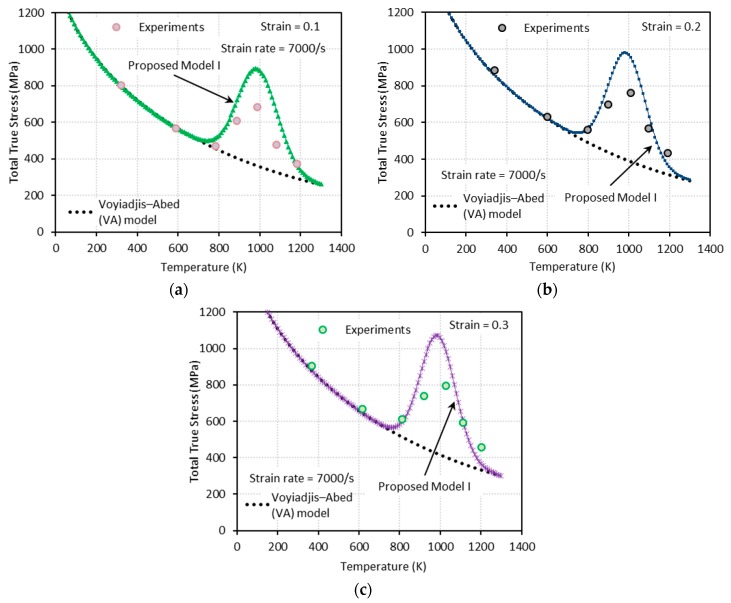
Comparisons between model predictions from the VA and proposed model I and experimental data from [1] on the total true stress versus temperature responses at (**a**) ε=0.1, (**b**) ε=0.2, and (**c**) ε=0.3. Dynamic loading with $\dot{\varepsilon }=7000\;s^{-1}$ is applied.

Figure 9, Figure 10, Figure 11 and Figure 12 show the true stress-temperature responses calculated by the VA model and PM I and their comparison with experimental results for the corresponding levels strain and applied strain rate. The VA model fails to predict (bell-shaped) hardening caused by DSA in all cases, whereas the PM I demonstrates its ability to capture the DSA effect in quasi-static loading cases (Figure 9 and Figure 10). When it comes to dynamic loading cases (Figure 11 and Figure 12), the PM I also fails to capture the bell-shaped hardening.

#### 3.2.2. Proposed Model II (PM II)

The function presented in Equation (21), along with Equations (22)–(24), is used for the PM II to model the DSA effect. The material parameters used for the PM II are summarized in Table 2.

The final form of the DSA-induced flow stress $\sigma_{D}$ for PM II can be expressed as follows:

(29) $$\sigma_{D}{}_{PM\;II}\left(\varepsilon_{p},\;{\dot{\varepsilon }}_{p},\;T\right)=\left[\left({\bar{a}}_{D}\ln\frac{{\dot{\varepsilon }}_{p}}{\dot{\zeta }}+{\overset{=}{a}}_{D}\right)\varepsilon_{p}^{n_{3}}\right]\exp\left[-{\left\{\frac{T-\frac{T_{1}}{\ln\frac{{\dot{\varepsilon }}_{p}}{\dot{\zeta }}-\eta \ln\frac{\varepsilon_{p}}{\varepsilon_{p}^{0}}}}{\frac{T_{2}}{\ln\frac{{\dot{\varepsilon }}_{p}}{\dot{\zeta }}-\eta \ln\frac{\varepsilon_{p}}{\varepsilon_{p}^{0}}}}\right\}}^{2}\right].$$

**Figure 13:**
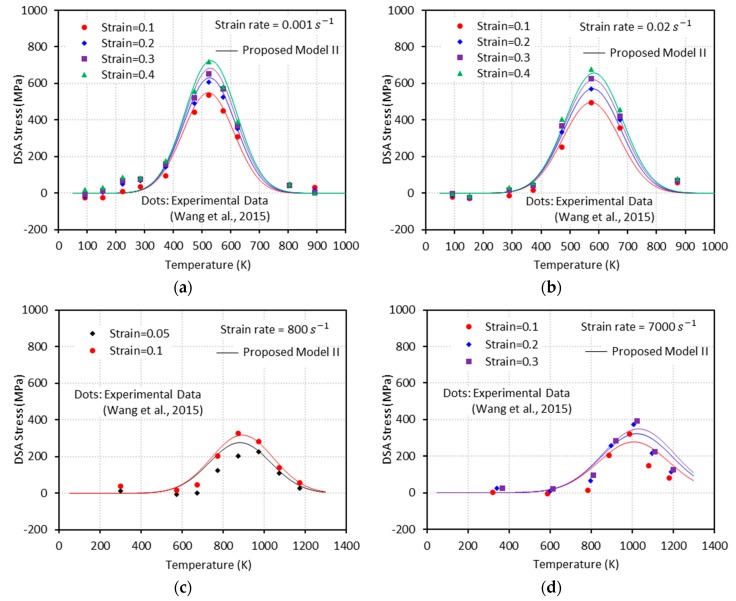
The DSA-induced flow stress versus temperature curves from the experiments [1] and the proposed model II with (**a**) $\dot{\varepsilon }=0.001\;s^{-1}$, (**b**) $\dot{\varepsilon }=0.02\;s^{-1}$, (**c**) $\dot{\varepsilon }=800\;s^{-1}$, and (**d**) $\dot{\varepsilon }=7000\;s^{-1}$.

The bell-shaped DSA-induced hardening versus temperature graphs are shown in Figure 13 at the designated strain levels. PM II is found able to capture the experimental measurements under quasi-static loading, cases (a) and (b), and dynamic loading, cases (c) and (d). In addition, unlike the PM I, the inversion point is not observed.

**Figure 14:**
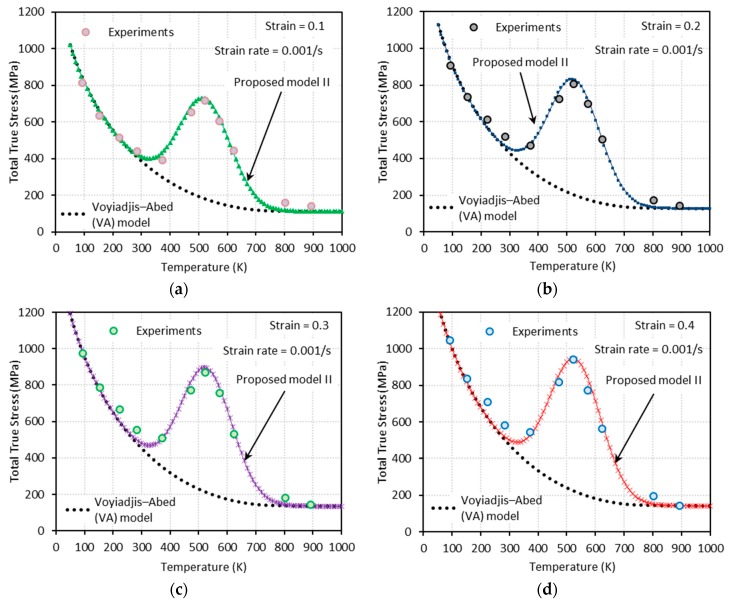
Comparisons between model predictions from the VA and proposed model II and experimental data from [1] on the total true stress versus temperature responses at (**a**) ε=0.1, (**b**) ε=0.2, (**c**) ε=0.3, and (**d**) ε=0.4. Quasi-static loading with $\dot{\varepsilon }=0.001\;s^{-1}$ is applied.

**Figure 15:**
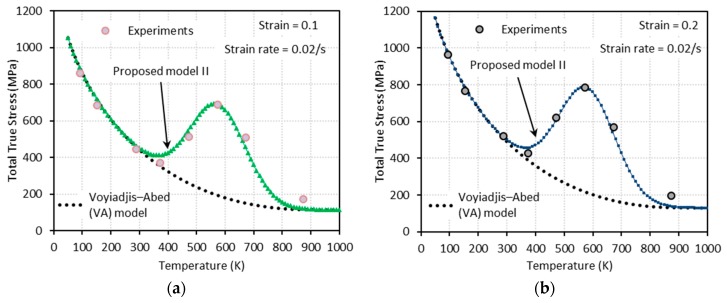

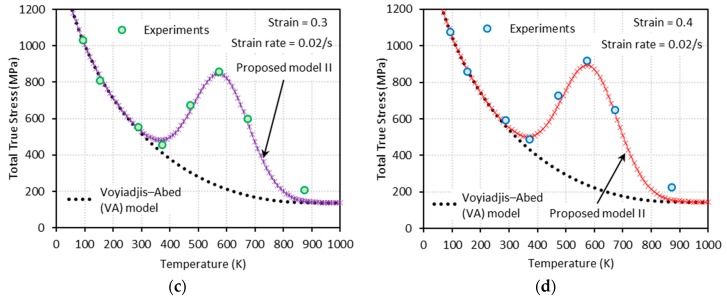
Comparisons between model predictions from the VA and proposed model II and experimental data from [1] on the total true stress versus temperature responses at (**a**) ε=0.1, (**b**) ε=0.2, (**c**) ε=0.3, and (**d**) ε=0.4. Quasi-static loading with $\dot{\varepsilon }=0.02\;s^{-1}$ is applied.

**Figure 16:**
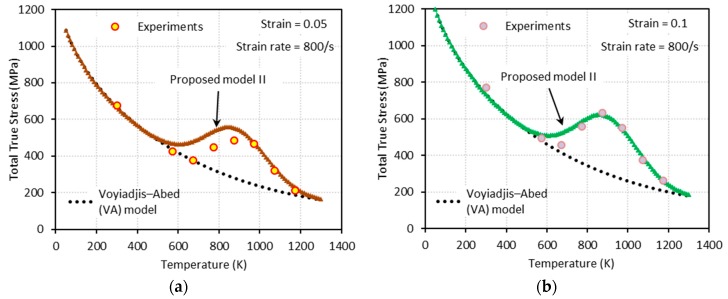
Comparisons between model predictions from the VA and proposed model II and experimental data from [1] on the total true stress versus temperature responses at (**a**) ε=0.05 and (**b**) ε=0.1. Dynamic loading with $\dot{\varepsilon }=800\;s^{-1}$ is applied.

**Figure 17:**
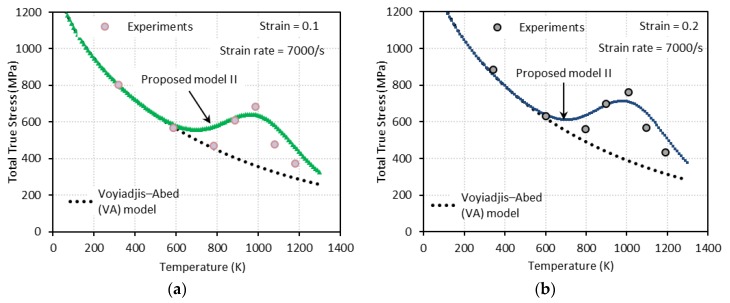

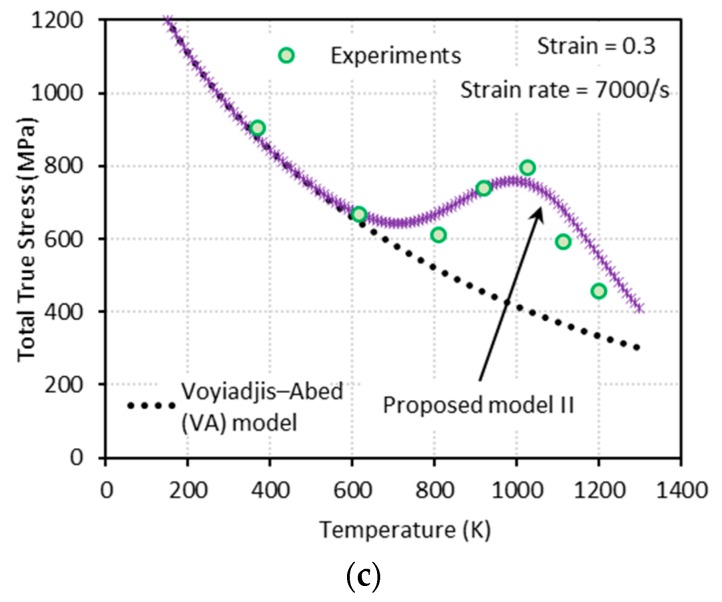
Comparisons between model predictions from the VA and proposed model II and experimental data from [1] on the total true stress versus temperature responses at (**a**) ε=0.1, (**b**) ε=0.2, and (**c**) ε=0.3. Dynamic loading with $\dot{\varepsilon }=7000\;s^{-1}$ is applied.

Figure 14, Figure 15, Figure 16 and Figure 17 show the total true stress-temperature responses calculated by the VA and PM II models along with the experimental results for the corresponding strain levels and applied strain rate. Unlike PM I, PM II demonstrates its ability to capture the DSA effect in all cases including dynamics loading.

#### 3.2.3. Strain Rate Effect on the DSA Stress

**Figure 18:**
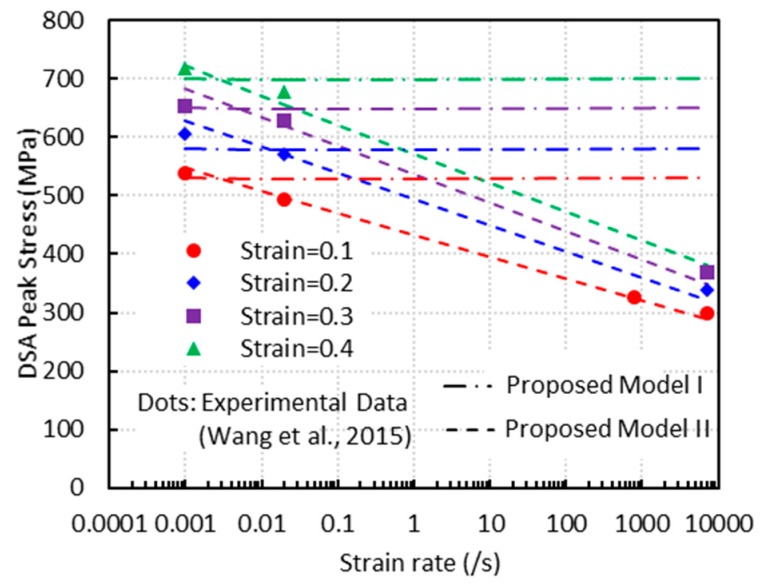
Variations of the DSA peak stress with strain rate at different strain levels.

As revealed in the experiments published by [1] (Figure 8, Figure 13 and Figure 18), the height of bell-shaped DSA stress (referred to as DSA peak stress in this section) decreases with strain rate for all strain levels. This observation is consistent with previous works by Nandy et al. [27] and Peng et al. [28].

The variations of the DSA peak stress with strain rate at different levels of strain are plotted in Figure 18. PM I and PM II are compared with the experimental measurements. As mentioned earlier, the height of the DSA stress is determined by the term $a_{D}$. In PM I, this term is independent of the strain rate, i.e., $a_{D}=k\varepsilon_{p}^{n}$, therefore it is unable to capture the trend of decreasing as shown in Figure 18. PM II, on the other hand, can describe this trend and capture the experimental data due to the linear dependence of $a_{D}$ on the logarithmic strain rate, i.e., $a_{D}={\bar{a}}_{D}\ln\frac{\dot{\varepsilon }}{\dot{\zeta }}+{\overset{=}{a}}_{D}$.

## 4. Comparison between the Model Predictions (VA Model, Proposed Model I, and Proposed Model II) and the Experimental Measurements

The experimental true stress-true strain data are investigated in this section. The model proposed by [15] is not expected to capture the stress-strain curves when DSA is active. To prove the ability of PM II to accurately predict DSA, it is compared to experimental measurements. PM I is also considered here for comparison.

**Figure 19:**
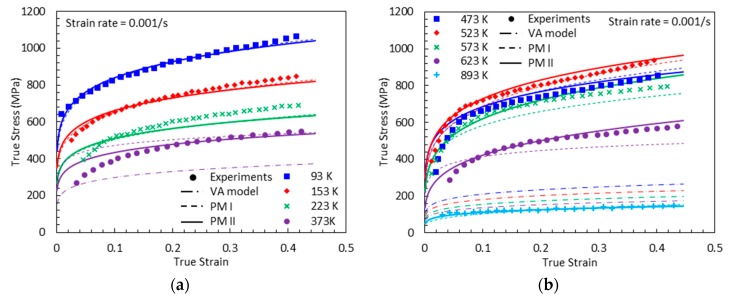
True stress-true strain curves from experimental measurement [1], predictions by the VA model, proposed model (PM) I, and PM II with $\dot{\varepsilon }=0.001\;s^{-1}$: (**a**) T=93, 153, 223 and 373 K (**b**) T=473, 523, 573, 623 and 893 K.

**Figure 20:**
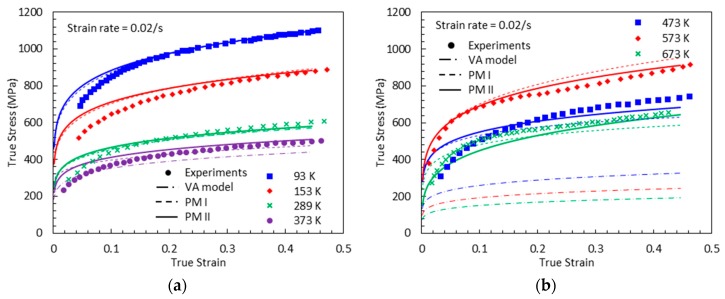
True stress-true strain curves from experimental measurement [1], predictions by the VA model, PM I, and PM II with $\dot{\varepsilon }=0.02\;s^{-1}$: (**a**) T=93, 153, 289 and 373 K (**b**) T=473, 573 and 673 K.

**Figure 21:**
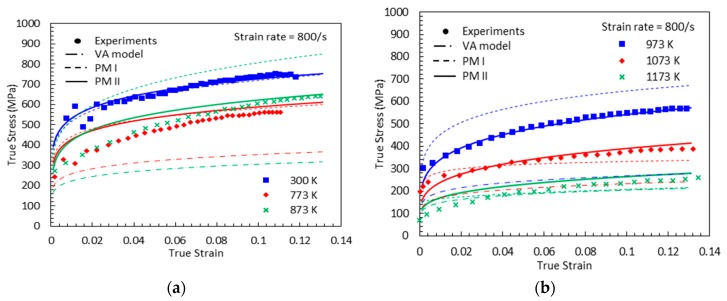
True stress-true strain curves from experimental measurement [1], predictions by the VA model, PM I, and PM II with $\dot{\varepsilon }=800\;s^{-1}$: (**a**) T=300, 773 and 873 K (**b**) T=973, 1073 and 1173 K.

**Figure 22:**
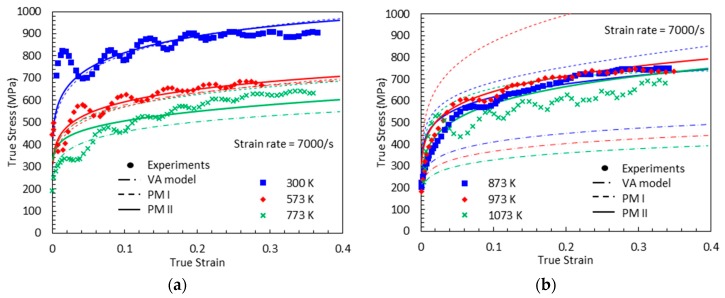
True stress-true strain curves from experimental measurement [1], predictions by the VA model, PM I, and PM II with $\dot{\varepsilon }=7000\;s^{-1}$: (**a**) T=300, 573 and 773 K (**b**) T=873, 973 and 1073 K.

Figure 19, Figure 20, Figure 21 and Figure 22 compare the true stress-true strain curves predicted by the different models considered with the experimental data measured by [1] for four different strain rates: $\dot{\varepsilon }=0.001\;s^{-1}$, $\dot{\varepsilon }=0.02\;s^{-1}$, $\dot{\varepsilon }=800\;s^{-1}$, and $\dot{\varepsilon }=7000\;s^{-1}$. The VA model is not able to capture the stress-strain responses when DSA is active. The next noticeable thing is that PM I overestimates the stress values under the active DSA at dynamic loading, for instance $\left(T,\;\dot{\varepsilon }\right)=\left(873\;K,\;800\;s^{-1}\right)$, $\left(973\;K,\;800\;s^{-1}\right)$, $\left(973\;K,\;7000\;s^{-1}\right)$. The reason is that the strain-rate effect is not considered in PM I when describing the magnitude of DSA-induced hardening. Moreover, PM II shows a good agreement with the experimental data in all cases.

**Figure 23:**
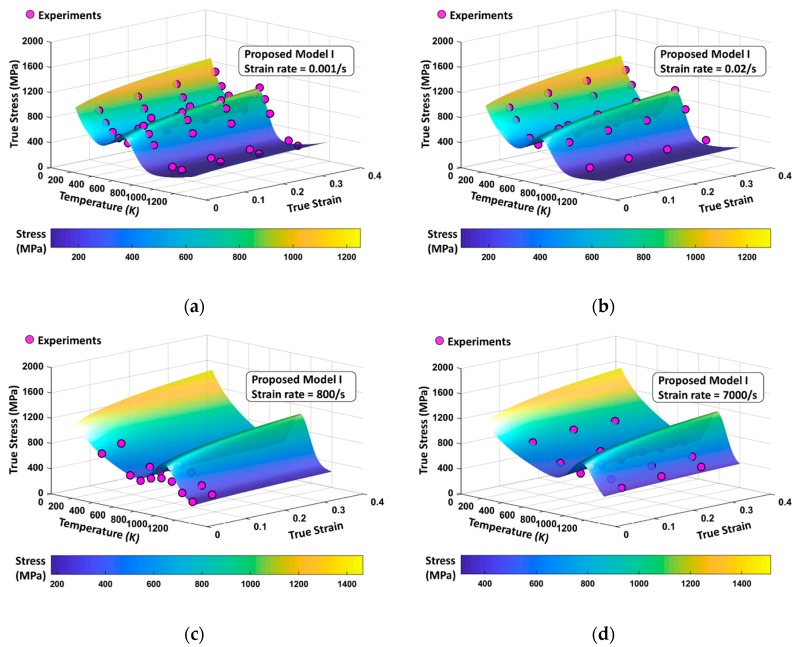
PM I flow stress surfaces according to variation of temperature and strain level with (**a**) $\dot{\varepsilon }=0.001\;s^{-1}$, (**b**) $\dot{\varepsilon }=0.02\;s^{-1}$, (**c**) $\dot{\varepsilon }=800\;s^{-1}$, and (**d**) $\dot{\varepsilon }=7000\;s^{-1}$. The experimental data are from [1].

**Figure 24:**
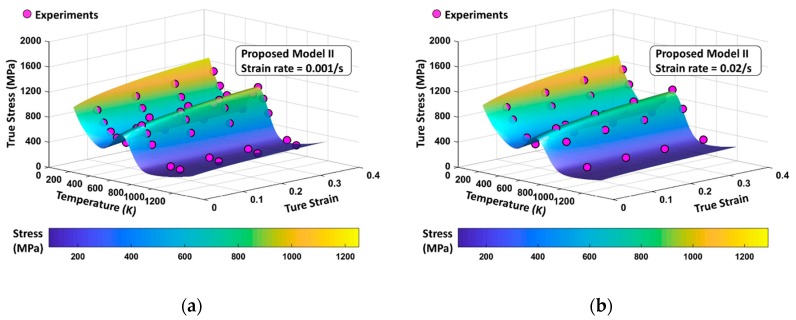

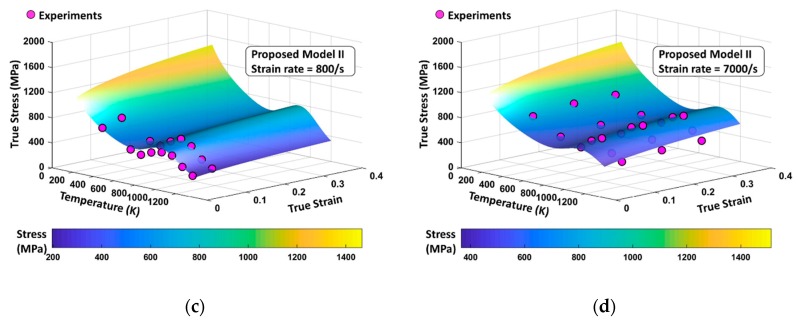
The PM II flow stress surfaces according to variation of temperature and strain level with (**a**) $\dot{\varepsilon }=0.001\;s^{-1}$, (**b**) $\dot{\varepsilon }=0.02\;s^{-1}$, (**c**) $\dot{\varepsilon }=800\;s^{-1}$, and (**d**) $\dot{\varepsilon }=7000\;s^{-1}$. The experimental data are from [1].

The total flow stress surfaces from PM I and PM II for four different strain rates ($\dot{\varepsilon }=0.001\;s^{-1}$, $0.02\;s^{-1}$, $800\;s^{-1}$, and $7000\;s^{-1}$) are shown in Figure 23 and Figure 24 ranging in temperatures from 0 K to 1200 K and in strains from 0.05 to 0.4. Dots indicate the experimental results and they are shown to be located mostly near the surfaces in both models when quasi-static loading is applied. Under dynamic loading, PM II shows a good agreement with experiments, whereas PM I provides overestimated values, especially near the peak of DSA-induced stress.

## 5. Strain Rate Sensitivity

In general, DSA is associated with spatio-temporal instabilities, and the strain rate sensitivity, quantified by the strain rate sensitivity exponent ($m=\partial \log\sigma /\partial \log\dot{\varepsilon }$) is a key point to study instabilities as reported in [29]. The negative strain rate sensitivity due to DSA may trigger instabilities more quickly in comparison with a material having a positive strain rate sensitivity. In general, the process is related to a competition between strain rate sensitivity, hardening, and temperature sensitivity as discussed in [29].

**Figure 25:**
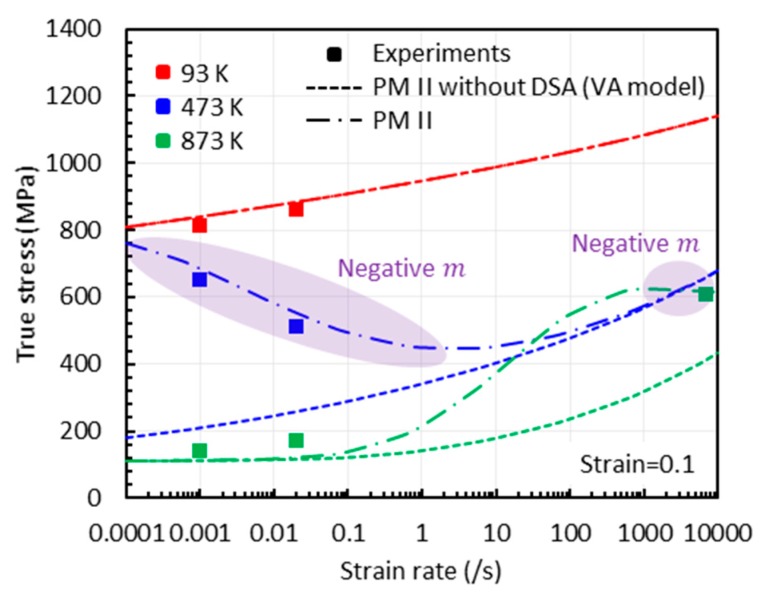
True stress versus strain rate graphs with three temperatures (93 K, 473 K, and 873 K ) at ε=0.1. The experimental data are from [1].

The variation of the total true stress with strain rate from PM II with and without DSA is plotted in Figure 25. PM II without the DSA component (i.e., VA model) always results in a positive strain-rate sensitivity (m>0) while PM II causes a negative strain-rate sensitivity acting only in some ranges of $\left(T,\dot{\varepsilon }\right)$. For this reason, the material will behave in two different ways, with a positive or negative strain-rate sensitivity, depending on the domain.

## 6. Conclusions

In this work, a systematic theoretical modeling of the plastic flow behavior of Q235B steel was performed over a wide range of strain rates and temperature. The characteristics of the bell-shaped hardening due to DSA in stress-temperature curves were investigated. In this work, a new mathematical expression along with a probability function was developed to accurately capture the experimental results. Findings of this study can be summarized as follows:

- Dynamic strain aging, which is characterized by the bell-shaped hardening in stress-temperature curves, appears under both quasi-static and dynamic loadings. As the strain rate increases, this bell-shaped hardening moves to elevated temperature region and the magnitude of hardening reduces.
- The VA model is not able to predict the bell-shaped hardening.
- The proposed model II shows an excellent agreement with the experimental results at both low and high strain rates, whereas the proposed model I fails to capture them at high strain rates.
- The negative strain rate sensitivity due to DSA is well captured by the proposed model II unlike the VA model.
- To be noted is the ease of incorporating the model formulation into existing algorithms for its use in finite element solvers [30,31,32,33,34,35,36,37].

## Figures and Tables

**Table 1:** Material parameters used in the Voyiadjis–Abed (VA) model for Q235B.

$Y_{a}\;\left(MPa\right)$	$B_{1}\;\left(MPa\right)$	$n_{1}\;\left(-\right)$	$Y_{d}\;\left(MPa\right)$	$B_{2}\;\left(MPa\right)$	$n_{2}\;\left(-\right)$	${\dot{\varepsilon }}_{p}^{0}\;\left(s^{-1}\right)$
0	166	0.18	100	1800	0.15	1.0
$\beta_{1}^{Y}$(1/K)	$\beta_{2}^{Y}$(1/K)	$\beta_{1}^{H}$(1/K)	$\beta_{2}^{H}$(1/K)	p (−)	q (−)	
$5.0\times 10^{-4}$	$4.7\times 10^{-5}$	$9.0\times 10^{-4}$	$5.5\times 10^{-5}$	0.51	1.65	

**Table 2:** Material parameters used in PM II for Q235B.

${\bar{a}}_{D}\;\left(MPa\right)$	${\overset{=}{a}}_{D}\;\left(MPa\right)$	$n_{3}\;\left(-\right)$	$\dot{\zeta }\;\left(s^{-1}\right)$	$T_{1}\;\left(K\right)$	$T_{2}\;\left(K\right)$	η (−)	$\varepsilon_{p}^{0}\;\left(-\right)$
−27	10	0.20	$6.5\times 10^{10}$	−17,000	−4100	−0.35	1.0
